# Traditional Indigenous medicine in North America: A scoping review

**DOI:** 10.1371/journal.pone.0237531

**Published:** 2020-08-13

**Authors:** Nicole Redvers, Be’sha Blondin

**Affiliations:** 1 Department of Family & Community Medicine, School of Medicine and Health Sciences, University of North Dakota, Grand Forks, North Dakota, United States of America; 2 Arctic Indigenous Wellness Foundation, Yellowknife, Northwest Territories, Canada; Southern Cross University, UNITED STATES

## Abstract

**Background:**

Despite the documented continued use of traditional healing methods, modalities and its associated practitioners by Indigenous groups across North America, it is presumed that widespread knowledge is elusive amongst most Western trained health professionals and systems. This despite that the approximately 7.5 million Indigenous peoples who currently reside in Canada and the United States (US) are most often served by Western systems of medicine. A state of the literature is currently needed in this area to provide an accessible resource tool for medical practitioners, scholars, and communities to better understand Indigenous traditional medicine in the context of current clinical care delivery and future policy making.

**Methods:**

A systematic search of multiple databases was performed utilizing an established scoping review framework. A consequent title and abstract review of articles published on traditional Indigenous medicine in the North American context was completed.

**Findings:**

Of the 4,277 published studies identified, 249 met the inclusion criteria divided into the following five categorical themes: General traditional medicine, integration of traditional and Western medicine systems, ceremonial practice for healing, usage of traditional medicine, and traditional healer perspectives.

**Conclusions:**

This scoping review was an attempt to catalogue the wide array of published research in the peer-reviewed and online grey literature on traditional Indigenous medicine in North America in order to provide an accessible database for medical practitioners, scholars, and communities to better inform practice, policymaking, and research in Indigenous communities.

## Introduction

The United Nations Declaration on the Rights of Indigenous Peoples (UNDRIP) was a pivotal document for the world’s Indigenous Peoples [1]. In addition to being quoted in numerous policy, research, and community initiatives since it was adopted, the declaration is now being used to evaluate the adequacy of national laws; for interpreting state obligations at the global level; and by some corporations, lending agencies, and investors in regards to resource and development opposition on Indigenous lands [2]. Article 24 of the declaration states that “Indigenous peoples have the right to their traditional medicines and to maintain their health practices, including the conservation of their vital medicinal plants, animals, and minerals” (UN document A/RES/61/295). The World Trade Organization has stated that “traditional medicine contributes significantly to the health status of many communities and is increasingly used within certain communities in developed countries. Appropriate recognition of traditional medicine is an important element of national health policy” [3].

The United Nation’s Economic and Social Council President in 2009, Sylvie Lucas, stated that “[t]he potential of traditional medicine should be fostered. … ‘We cannot ignore the potential of traditional medicine’ in the race to achieve the Millennium Development Goals and renew primary health care for those who lacked access to it … traditional medicine [is] a field in which the knowledge and know-how of developing countries was ‘enormous’—and that was a source of hope for improving the world’s health-care situation” [4].

In November 2008, member states of the World Health Organization (WHO) adopted the Beijing Declaration [5], where they recognized the role of traditional medicine in the improvement of public health and supported its integration into national health systems where appropriate [6]. The declaration also promotes improved education, research, and clinical inquiry into traditional medicine, as well as improved communication among health-care providers [6].

Research into some types of complementary and alternative medicine (CAM) practices has received large amounts of funding. For example, the US National Institute of Health has a division called the National Center for Complementary and Alternative Medicine (NCCAM), which in 2010 had a budget of US$128.8 million dollars [7]. Before the Beijing Declaration, sixty-two countries had national institutes for traditional medicine as of 2007, up from twelve in 1970 [4]. Despite this, there has been a complete lack of acknowledgement of the Indigenous traditional knowledge (TK) currently being used in many CAM professions. In some cases, there has been direct cultural appropriation of traditional medicine and practices by CAM or other biomedical groups in North America [8]. Although outdated, given the lack of scholarship in this area, a 1993 estimate put the total world sales of products derived from traditional medicines as high as US$43 billion [9]; however, only a tiny fraction of the profits were and are being returned to the Indigenous peoples and local communities from where these medicines were derived. In the early 1990s, it was estimated that “less than 0.001 per cent of profits from drugs developed from natural products and traditional knowledge accrue to the traditional people who provided technical leads for research” [10].

So, despite some progress on a global level in CAM research and practice, many Indigenous medicine systems around the world are still often given the back seat when it comes to both acknowledgement and practice within the conventional medical-care setting. The terms and attributes used for traditional medicine, such as ‘alternative’, translates into an epistemological discomfort regarding the identity of these medicines [11] that automatically sets a power differential from conventional care. In 2007, *The Lancet* published an article in which the authors stated, “[w]e now call on all health professionals to act in accordance with this important UN declaration of [I]ndigenous rights—in the ways in which we work as scientists with [I]ndigenous communities; in the ways in which we support [I]ndigenous peoples to protect and develop their traditional medicines and health practices; in our support and development of [I]ndigenous peoples’ rights to appropriate health services; and most importantly in listening, and in supporting [I]ndigenous peoples’ self-determination over their health, wellbeing, and development” [12].

In his 2008 dissertation, (Gus) Louis Paul Hill noted that there is a paucity of literature on Indigenous approaches to healing within Canada specifically, and little documentation and discussion of Indigenous healing methods in general [13]. With this, there is currently no formal Canadian (or US based) Indigenous health policy framework or national adopted policy on Indigenous traditional medicine [14,15], and no *broad* application and endorsement of Indigenous ways of achieving wellness markers that are self-determined in an already marginalized community (demonstrated by a lack of funding and accessibility to these services generally).

Despite this being an emerging scholarship area, with a clear lack of reflected national health policy, there is increasing evidence on the use of traditional Indigenous medicine in certain areas of need such as in substance abuse and addictions treatment [16–21]. When Canadian Indigenous communities were asked about the challenges currently facing their communities, 82.6% stated that the most common issue was alcohol and drug abuse [22] and that traditional medicine itself is a critically important part of Indigenous health [23], including in the support of addictions. Due to the often upstream, structural, and socio-political [24] factors driving substance abuse in addition to other health ailments in Indigenous communities, advancing co-production of treatment options such as utilizing traditional medicine that already fits into an Indigenous paradigm may ensure four key steps to wellness occur: decolonization, mobilization, transformation, and healing [25].

### The present study

Despite the documented continued use of traditional healing methods, modalities, and their associated practitioners by Indigenous groups across North America, widespread knowledge of this domain is presumed elusive among most Western-trained health professionals and systems. This despite the fact that the approximately 7.5 million Indigenous peoples who currently reside in Canada and the United States (US) are most often served by Western systems of medicine. There is current exploration in the literature on how cultural competency and safety impacts health disparities across diverse populations; however, there is little attention to how traditional Indigenous medicine systems fit into this practice area. Therefore, an account of the state of the literature is currently needed in this area of traditional Indigenous medicine to provide an accessible resource tool for medical practitioners, scholars, and communities in the North American context to better understand Indigenous traditional medicine in the context of current clinical care delivery and future policy making. In addition, having baseline literature on this topic area available for use in cultural safety training, and diversity and inclusion training on or off reservations, is warranted and in need.

Considering the paucity of accessible information on traditional Indigenous medicine, in addition to the lack of cohesive understanding on what traditional healing is within the Western context, the purpose of this present study is–

- to catalogue the current state of the peer-reviewed and online grey literature on traditional medicine in the North American context by identifying the types and sources of evidence available, and
- to provide an evidence-informed resource guide for medical practitioners, scholars, and communities to better inform “practice, policymaking, and research [26]” in Indigenous communities.

## Methods

The methodology for this scoping review was a mixed-methods approach (Western-Indigenous). The first four steps of the scoping review were conducted within a Western methodological approach as outlined by Pham et al. [27] and based on the framework outlined by Arksey and O’Malley [28] with subsequent recommendations made by Levac et al. [29] (i.e., (1) combining a broad research question with a clearly articulated scope of inquiry, (2) identifying relevant studies, (3) study selection, and (4) charting the data). For the fifth step, as outlined by Arksey and O’Malley [28], (i.e., (5) collating, summarizing, and reporting the results), we utilized a dominant Indigenous methodology that places a focus on personal research preparations with purpose, self-location, decolonization and the lens of benefiting the community [30–32]. Although this research process did include the Western conceptions of collating, summarizing, and reporting the results as per outlined and described by Arksey and O’Malley [28], there was a very clear intent of identifying ourselves, the authors, as being rooted within Indigenous communities, and within an Indigenous worldview. This meant that we were not able to *critique* or provide commentary to contradictory evidence found in the scoping review process, as it is not culturally appropriate to provide this type of analysis within the topic area of traditional medicine through an Indigenous worldview. As Saini points out, utilizing self-determined Indigenous methodologies is “critical to ensure Aboriginal research designs are not marginalized due to perceptions that they are somehow less valid or sophisticated than their counterparts” [33] at the community or systems level.

The sixth methodologic step in our scoping review, as advanced by Levac et al. [29], incorporates a consultation exercise involving key stakeholders to inform and validate study findings [26] and was done in parallel to all steps of the work. This was another mixed-method bridging step, where one Indigenous Elder who is considered a content expert in their respective community was utilized to ensure placement of the research in the Indigenous context despite the use of Western metrics for the data-collection portion of the work (as opposed to an academic or other institutional stakeholder). It must be noted that Indigenous Elders’ engagement with research is often solely for the purpose of benefiting their community [30–32]. This therefore creates a unique stakeholder engagement process that roots the research not to a specific Western-defined method or process but to a set of traditional Indigenous protocols (unwritten community directives defined through an Indigenous worldview) that must be followed to ensure uptake and acceptance of the work by Indigenous communities themselves. In essence, the ‘validation of study findings’ (as outlined by Levac et al. in their sixth methodologic step [29]) is not culturally malleable and needed to be changed to a process of reviewing the rules and parameters (i.e., traditional protocols) around how traditional medicine should be talked about in the context of research. The authors are both immersed in work with Indigenous communities and peoples and understands the importance of Indigenous research processes to move away from the conformity of Western notions of the scientific deductive process of new knowledge development, and instead to work towards providing space for the translational voices within Indigenous communities and peoples [34]. The review methodology was defined a priori.

### Eligibility criteria, procedures, and search terms

Only articles published in peer-reviewed academic journals or easily accessible online reputable organizational documents and dissertation works that were formally published (i.e., online grey literature) were included. No limits were put on the type of research conducted, whether qualitative, quantitative, commentary, or otherwise given the specific nature of the topic and the assumed limited studies available for review. Studies were included if they made reference to traditional medicine, or if they noted specific traditional medicine interventions or practitioners (i.e., sweat lodge, traditional healers, etc.). Ethnobotanical, plant physiology, and reviews of specific Indigenous plants were excluded from this scoping review as they were most often not based on the context of traditional medicine but the function and action of the plant itself. All studies up until June 29, 2020 were included in the review.

The authors did not specify a definition for ‘traditional medicine’ before selecting studies for this review, which was purposeful. There is currently a vast array of traditional medicine modalities, practices, and people across North America who may have varying definitions or interpretations of the terms and practice. This therefore required a broad inductive and immersive approach to allow the community of researchers in this area to provide their own definitions regionally, which therefore made an impact on the breadth of articles found. All the variants of the words for traditional medicine that were used to include articles were based on existing knowledge, a pre-screen of the available literature, and consultation with an Elder (see S1 Table and ‘title and abstract relevance screening’ section).

No restrictions were put on language for the initial search; however, only English language articles were considered for inclusion. This was also due to a complete lack of peer-reviewed articles written in an Indigenous language being noted in prior work, in addition to the prospective difficulties and budget needed to attain translation support. With a multitude of Indigenous languages in North America, there is an unfortunate lack of access to translators for projects such as these. Articles that were outside of the continental US and Canada were also excluded (i.e., Pacific Islanders, etc.), in addition to those from Mexico despite the proximity of traditional lands within and to the US. This was due to differences in traditional medicine practice and agents in those areas. Books and book reviews were not included due to the difficulty in verifying their content. North American Indigenous was defined to be First Nations, Inuit, Métis, American Indian, Alaskan Native or the respective Bands and Tribes within the region. As demographic terminology changes depending on the region of the continent, it was important to ensure complete capture of the eligible literature by utilizing both Canadian and US Indigenous terminologies. A two-stage screening process was used to assess the relevance of studies identified in the search as further outlined below.

The scoping review process and search terms were developed with the aid of a medical librarian (D.O) in discussion with the lead author (N.R.). The search was created in PubMed using a combination of key terms and index headings related to North American Indigenous peoples and traditional medicine (see S1 Table). The search was completed between December 27, 2018 and June 29, 2020 by searching the following databases with no limits on the start date, language, subject, or type: PubMed, EMBASE, PsycInfo, Elsevier’s Scopus, PROSPERO, and Dartmouth College’s Biomedical Library database due to the breadth of databases available in this library. In addition, manual searches of the following websites were completed: Indigenous Studies Portal, University of Saskatchewan [35]; National Collaborating Centre for Aboriginal Health [36]; the Aboriginal Healing Foundation’s archived website documents [37]; and the International Journal of Indigenous Health, which includes archives from the *Journal of Aboriginal Health*. Google Scholar was searched by inspecting the first two pages of results and then subsequently screening the next two pages if results were identified until no more relevant results had been found. The reference lists of randomly selected articles were manually searched with a “snowball” technique utilized to identify any further literature that may have been missed in the first search round until saturation of the search had been reached.

### Title and abstract relevance screening

A title and abstract relevance form was developed by the author (N.R) in a session during the Elder consultation (B.B), mainly by the *a priori* identification of the search terms used and as listed in the S1 Table. As the goal was to capture as much available literature on the subject as possible, the title and abstract review were non-restrictive other than the stated eligibility criteria and search terms noted above. The reviewer was not masked to the article authors or journal names as this was not a results-based review. Some article titles did not have an abstract available for review and were therefore included in the subsequent full review to better characterize the content relevance to the topic area. If there was a question on the relevance of an article for inclusion, the Elder was brought into the discussion (B.B) as the final authority for the decision on whether to proceed with inclusion.

### Data characterization, summary, and synthesis

After title and abstract screening, all the citations that were deemed relevant to the topic were kept in the scoping review database (S2 Table). All full text articles were obtained once identified as eligible; however, as the intent was not to provide critical review of the articles, they were not catalogued based on the completion of a full text article review. Instead, all articles were kept in the database from the title and abstract screening alone for the categorization process, ensuring that no judgement was placed on traditional medicine topics in keeping with an Indigenous methodological paradigm. Therefore a quality assessment procedure was not performed on the articles included in this scoping review as noted (e.g., Critical appraisal of qualitative research [38]) for a few reasons:

- The purpose of this review was to map the existing state of the literature on this topic and not to analyze the results of the included articles, and
- The vast array of formats and methodologies used in the Indigenous traditional medicine literature make the dominant Western metrics of validity simply not applicable to the current research purpose.

All citations found were compiled in a single Microsoft Excel 365 ProPlus spreadsheet. Coding of articles was done based on title and abstract review alone, with an Elder advisor to aid identification of categorical themes. Themes were based and developed by way of traditional knowledge (TK); however, it was noted in the synthesis process that there was often substantial overlap between themes. In these cases, a priority category was given for the ease of database creation which means that the categorical themes cannot be looked at as being black and white. Traditional Indigenous medicine is often very complex in its practice; however, an attempt was done to ease classification by assessing for the most discussed or most focused research topic(s) in each article.

## Results

Due to the substantial overlap of search terms used for traditional medicine in other disciplines (i.e., *traditional medicine* can be the term used from the Indigenous perspective or from the Western perspective), the initial search yielded thousands of articles.

**Fig 1:**
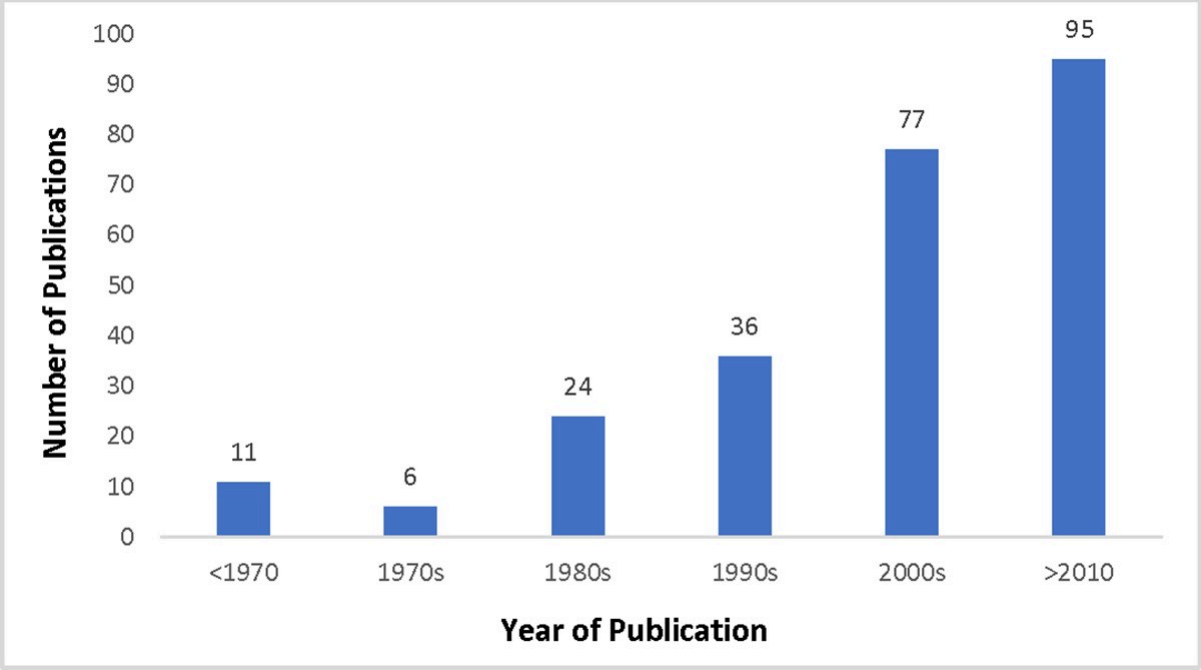
Trends in the number of articles identified in the scoping review over time.

Based on a review of the title and abstracts, 249 articles met the criteria for inclusion (see S2 Table for the full database of articles included). A full article review was conducted when the initial screen left questions about the relevance of the research for inclusion. Broad inclusion was purposeful, as by ensuring a wide capture of the literature was categorized, future research and program needs have a more complete database to pull information from. Articles ranged in date from the earliest year of publication, being in 1888, to the most recent publication, being in 2020 (Fig 1). Sixty two percent of the articles were published prior to 2009 (n = 154) with the average year of publication being 2001.

**Fig 2:**
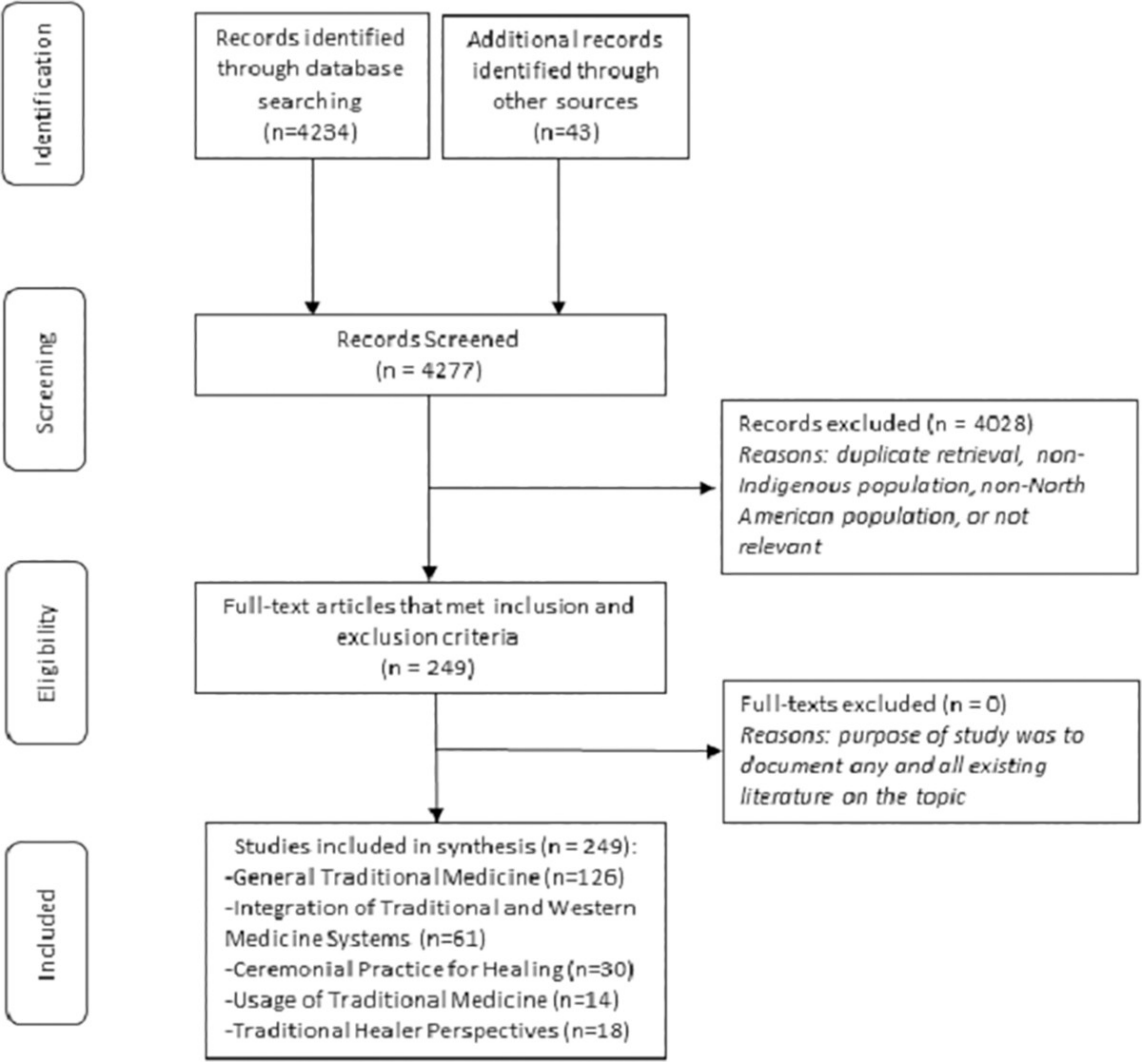
Adapted PRISMA diagram.

There were five overlapping categorical themes that emerged in the review including: General traditional medicine, integration of traditional and Western medicine systems, ceremonial practice for healing, usage of traditional medicine, and traditional healer perspectives. Fig 2 summarizes the selection process and findings.

### General traditional medicine

There were 126 articles identified for this category with the majority of the publications being from 2009 and earlier (75%, n = 95). Thirty-three of the articles were based in Canada, one was based in both Canada and the US, and the remaining ninety-two were based in the US alone. The publication dates for articles spanned a wide time period between 1888 and 2020 (average year of publication was 1997), with the majority being commentary or qualitative in nature.

In the review of this category, it became clearly evident that the terms or conceptualizations applied to traditional medicine or its variants (i.e., traditional healing, Native American healing, etc.) were very generalized. Specifically, the general research topics ranged from trying to answer the question of what is traditional medicine [13,39–41], to asking questions on the efficacy and acceptance of traditional medicine [42–44], to the applicability of traditional medicine with specific disease states [45–47], in addition to stories of healing by recipients of traditional medicine practice or approaches [48,49].

According to Alvord and Van Pelt, traditional medicine is described in the Navajo culture as a medicine that is performed by a *hataalii*, which is someone who sees a person not simply as a body, but as a whole being with body, mind, and spirit seen to be connected to other people, to families, to communities, and even to the planet and universe [50]. In helping to clarify the intent and purpose for utilizing a traditional Indigenous medicine approach, Hill describes it as “the journey toward self-awareness, self-knowledge, spiritual attunement and oneness with Creation” and “the lifelong process of understanding one’s gifts from the Creator and the embodiment of life’s teaching that [an] individual has received” [13]. The traditional medicine practitioner’s role in the healing process has been described as their being an instrument, a helper, the worker, the preparer, the doer in the healing process with the work using the “medicines” being slow, careful, respectful, and embodying a sense of humility [51].

Also of note in this section of articles, was the subtle distinction between the terms ‘traditional healing’ compared to the actual using of ‘traditional medicines’. The core of ‘traditional healing’ was said to be or attaining spiritual ‘connectedness’, in which there were many stated ways for developing this in order to have a strong physical body and mind [52]. In essence, this ‘connectedness’ could be with or without the actual use of what we would call a ‘medicine’ in Western terms achieved instead through being in harmony with the natural environment, through fasting, prayer, or meditation, or through the use of actual ‘traditional medicines’ that could include plant- and herb-based medicines [52].

Quantitative data analysis within the general traditional medicine category of articles was rarely performed. When quantitative analysis was performed, it was usually done in a mixed method format that utilized survey tools alongside qualitative approaches (e.g., interviews, focus groups) [53–55]. For example, a mixed methods study by Mainguy et al., found that the level of spiritual transformation achieved through interaction with traditional healers was associated with a subsequent improvement in medical illness in 134 of 155 people (*P* < .0001), and that this association exhibited a dose-response relationship [55]. In another mixed-methods study by Marsh et al., a 13-week intervention with “Indigenous Healing and Seeking Safety” in 17 participants demonstrated improvement in trauma symptoms, as measured by the TSC-40, with a mean decrease of 23.9 (SD = 6.4, p = 0.001) points, representing a 55% improvement from baseline [53]. Furthermore, in this study all six TSC-40 subscales demonstrated a significant decrease (i.e., anxiety, depression, sexual abuse trauma index, sleep disturbance, dissociation, and sexual problems) [53].

It was clear from the review of articles in this category that a large number of the articles were written from an observational or commentary perspective by non-Indigenous scholars (e.g., anthropologic perspectives) [42,56]. Those written more than twenty years ago often had titles or content that would not be considered culturally appropriate in today’s scholarly work. For example, an article by Walter Vanast from 1992 was titled, “‘Ignorant of any Rational Method’: European Assessments of Indigenous Healing Practices in the North American Arctic” [57]. Considerations for the issue of quality and accuracy in this body of literature will be addressed in the discussion section of this paper.

### Integration of traditional and Western medicine systems

A total of 61 articles in this category were reviewed, with publication dates ranging from the year 1974 to the year 2019 (average year of publication was 2006). Sixteen percent (n = 10) of these articles were from nursing journals, and 39% (n = 24) were articles from mental health and/or substance abuse journals. Of the total number of articles in this section, 61% (n = 37) were based in the US, with the remaining being from Canada (n = 24).

Articles in this category fell into overlapping subsets within the overarching theme of the integration of traditional Indigenous medicine systems with Western medicine systems. There were articles specifically calling for physicians and other healthcare providers to better collaborate with traditional healers [58,59], and also calls for health “systems” to better coordinate and work with Indigenous medicine systems and associated practitioners [60–62]. Some of the articles focused on cultural accommodations, and awareness and attitudes in medical settings towards traditional medicine and healers [63–65]. Lastly, a number of articles reviewed existing medical environments, practitioners, and facilities that had either piloted or fully integrated traditional and Western medical care under the same roof or practice [24,66–69].

The integration of traditional medicine into existing medical education environments was showcased through a residency training program as described by Kessler et al. [70]. In 2011, the University of New Mexico Public Health department and their General Preventive Medicine Residency Program in the United States started to integrate traditional healing into the resident training curriculum with full implementation completed by 2015. An innovative approach was used in the teaching delivery by utilizing a compendium of training methods, which included learning directly from traditional healers and direct participation in healing practices by residents [70]. The “incorporation of this residency curriculum resulted in a means to produce physicians well trained in approaching patient care and population health with knowledge of culturally based health practices in order to facilitate healthy patients and communities” [70].

Other articles in this section described the role of nurses in advocating for Indigenous healing programs and treatment. In research by Hunter et al., healing holistically can be said to match the time-honored values seen in the nursing profession: caring, sharing, and empowering clients [71]. Participant observations demonstrated that health centers could support progression along a cultural path by providing traditional healing with transcultural nurses acting as lobbyists for culturally sensitive health programs directed by Indigenous peoples [71]. This need for advocacy and awareness building on traditional ways of healing were emphasized throughout this category of articles.

According to Joseph Gone, “Lakota doctoring [traditional healing] remains highly relevant for wellness interventions and healthcare services even though it is not amenable in principle to scientific evaluation” [72]. In reference to Indigenous healing practices in general, Gone states that in Indigenous settings “we already know what works in our communities” and this claim seems “to reflect the vaunted authority of personal experience within Indigenous knowledge systems [72].

Some scholars noted the potential harms of not moving towards a respectful dialogue between the two systems of medicine (i.e., Western and Indigenous). A noted article by David Baines, an Indigenous physician from the Tlingit/Tsimshian tribe in Southeast Alaska, describes one of his patients who had metastatic lung cancer [73]. The patient had an oncologist but also went to a traditional healer to help deal with the pain she was having [73]. When the patient told the oncologist she was seeing a traditional healer, the oncologist got angry and wanted to know why she wanted to see a “witch doctor” [73]. The patient was offended and angry and refused to go back to the oncologist. She ended up dying a very painful death. Dr. Baines noted that it is important to remember we have the same goal—a healthy patient [73].

### Ceremonial practice for healing

Thirty articles were identified for this category. Important sub-categories became apparent in the review, including sweat lodge ceremonies (n = 15), traditional tobacco ceremonies and use (n = 6), birth and birthplace as a ceremony (n = 2), puberty ceremonies (n = 4), and using ceremony as a model for healing from a relative’s death or from trauma (n = 3). There were only eight Canadian studies published in this category, with the majority being based in the US (n = 22).

Sweat lodge ceremonies (SLC) have been practiced by many Indigenous nations since ancient times. SLCs are used as a process of honoring transformation and healing that is central to many Indigenous traditionalisms [74]. Gossage et al. examined the role of SLCs in the treatment for alcohol use disorder in incarcerated people [75]. The Dine Center for Substance Abuse Treatment staff utilized SLCs as a specific modality for jail-based treatment and analyzed its effect on a number of parameters. Experiential data was collected from 123 inmates after SLCs with several cultural variables showing improvement [75]. Gossage et al. also reported results from a similar prior study that analysed data for 100 inmates who participated in SLCs [75]. The research found that incarceration recidivism rates for those SLC participants was only 7% compared with an estimated 30–40% for other inmates who did not participate in such ceremonies [75]. Another study by Marsh et al., gathered qualitative evidence about the impact of the SLC on participants in a trauma and substance-abuse program and reported an increase in spiritual and emotional well-being that participants said was directly attributable to the ceremony [76].

Much of the existing literature on ceremonial tobacco focuses on either the perception of usage or the usage in general by Indigenous peoples in the region examined. In research done by Struthers and Hodge, six Ojibwe traditional healers and spiritual leaders described the sacred use of tobacco [77]. Interviews with these traditional healers confirmed that “sacred tobacco continues to play a paramount role in the community and provides a foundation for the American Indian Anishinabe or Ojibwe culture. They reiterated that using tobacco in the sacred way is vital for the Anishinabe culture [as] tobacco holds everything together and completes the circle. If tobacco is not used in a sacred manner, the circle is broken and a disconnect occurs in relation to the culture” [77].

The exploration of ceremonies surrounding birth and the relationship that is created through birth practices were outlined in a few studies reviewed for this category [78,79]. Ceremony was referred to in this context as the practice of what can be considered “rituals of healing”, noting that pregnancy itself “is carrying sacred water” [78]. As Rachel Olson points out, “[b]ringing people “back” to practicing ceremonial ways is seen as a healing process from the trauma encountered by First Nations peoples in Canada, as well as a way to both maintain our connection to the land and water, and to keep that same land and water safe for future generations. The implication in this is that by restoring our connection to the land through ceremony, other structural issues will again come into balance” [78].

### Usage of traditional medicine

Data collection was completed in reservation and urban Indigenous communities to determine the usage rates of traditional medicine by Indigenous peoples. There was a total of 14 articles published on this topic, which included over 650 participants combined who completed surveys or interviews. Five studies were completed in Canada, and the remaining were completed in the United States (n = 9). Seventy-nine percent of the studies were published prior to 2009 (n = 11). The average year of publication was 2002 with publication dates ranging from 1988 to 2017. Rates of usage of both traditional medicines and traditional healers varied per region. Relevant findings are summarized in Table 1.

**Table 1:** Included studies in the category “Usage of traditional medicine”.

Author	Year	Location	N	Key Findings
Garro [80]	1988	Ojibwe community, Manitoba	35	Four informants stated they did not use traditional medicine while the majority reported successful treatments with most reporting at least three episodes of traditional medicine treatment.
Waldram [81]	1990	Saskatoon, Saskatchewan	226	19% had a past consultation with a traditional healer. 27% had used herbal medicines or sweetgrass with the majority being within the last three months. 100% of those that had a past consultation with a traditional healer had an Indigenous language as their first language.
Garro [82]	1991	Anishinaabe reserve community, Manitoba	468	17% of cases involved visits to medicine men to request a diagnosis. Visits to an Anishinaabe healer occurred in 21% of the cases. 7% of visits to medicine men took place without consulting physicians, either prior to or after the visit to the medicine man. Of the 61 households visited, 62% reported visits to medicine men during the case collection period. In all but a few cases, treatment by medicine men was viewed positively by the reporting households for the specific illness condition in question.
Marbella et al. [83]	1998	Urban Indian Health Service clinic in Milwaukee, Wisc.	150	38% of the patients see a healer, and of those who do not, 86% would consider seeing one in the future. Sweat lodge ceremonies, spiritual healing, and herbal remedies were the most common treatments. More than a third of the patients seeing healers received different advice from their physicians and healers. The patients rate their healer’s advice higher than their physician’s advice 61.4% of the time. Only 14.8% of the patients seeing healers tell their physician about their use.
Kim et al. [84]	1998	Navajo Reservation-Indian Health Service Hospital	300	62% of Navajo patients had used Native healers and 39% used Native healers on a regular basis.
Wyrostok et al. [85]	2000	Canadian First Nation Students	99	Over 80% of respondents affirmed there interest in learning more about Native healing. Participants strongly supported traditional healing practices as something that should not be forgotten. 80.8% of participants reported at least some previous experiences with specific traditional healing practices.
Buchwald et al. [86]	2000	Urban primary care program, The Seattle Indian Health Board	869	70% of urban American Indian/Alaskan Native patients in primary care often used traditional health practices and use was strongly associated with cultural affiliation.
Gurley et al. [87]	2001	Vietnam veterans in the reservation communities of the Southwest and Northern Plains	621	17.1% of the Southwest reservation respondents and 4.7% of the Northern Plains reservation respondents saw a traditional healer for a physical health problem. 18.5% of the Southwest reservation respondents and 5.0% of the Northern Plains reservation respondents saw a traditional healer for a mental health problem.
Van Sickle et al. [88]	2003	Navajo families with asthmatic members	35	46% of families had previously used traditional healing; however, only 29% sought traditional healing for asthma.
Novins et al. [89]	2004	Enrolled members of a Northern Plains or a Southwest tribe	2595	Traditional healing provided a greater proportion of care for psychiatric (63.8% in the Southwest, 36.1% in the Northern Plains) than for physical health problems (44.6% and 13.9%). Compared with their counterparts in the Northern Plains, service users from the Southwest were more likely to use traditional healing only (22.0% vs. 3.5%) for physical health problems.
Cook [90]	2005	Mi'kmaq First Nation community health clinic	100	66% of respondents had used Mi’kmaq medicine, and 92.4% of these respondents had not discussed this with their physician. Of those who had used Mi’kmaq medicine, 24.3% use it as first-line treatment when they are ill, and 31.8% believe that Mi’kmaq medicine is better overall than Western. Even among patients who have not used Mi’kmaq medicine, 5.9% believe that it is more effective than Western medicine in treating illness.
Moghaddam et al. [91]	2013	Urban Indian health and community center (AIHFS), Detroit	389	Analyses indicated that experiences of discrimination in healthcare settings were significantly associated with participation in traditional healing. Nearly half of the Detroit sample (48%, n = 185) had used traditional services.
Greensky et al. [92]	2014	Fond du Lac Band Reservation	21	66% of participants described using traditional practices for healing and pain relief; 90% of individuals interviewed endorsed inclusion of traditional health practices into their medical care.
George et al. [52]	2017	Two First Nations communities in Ontario	613	About 15% of participants used both traditional medicines and healers, 15% used traditional medicines only, 3% used a traditional healer only, and 63% did not use either. Of those who did not use traditional healing practices, 51% reported that they would like to use them. Common reasons for not using traditional practices were not knowing enough about them, and not knowing how to access or where to access them.

Overall, the perception of traditional medicine amongst Indigenous people were positive. Several studies noted that access was an issue for many respondents who had the stated desire to use traditional medicine or see a traditional healer but did not know where to go for this support or treatment.

### Traditional healer perspectives

The viewpoints of traditional healers themselves are an important contribution to this research topic. There were 18 studies that elicited the perspectives from Elders and traditional healers ranging in dates of publication between 1993 and 2019 (average year of publication was 2011). Twelve studies were either fully or partially based in the US, with nine articles published in either nursing or mental health related journals.

Moorehead et al. describe discussions held with a group of traditional healers on the possibilities and challenges of collaboration between Indigenous and conventional biomedical therapeutic approaches [93]. The participants recommended the implementation of cultural programming, the observance of mutuality and respect, the importance of clear and honest communication, and the need for awareness of cultural differences as a unique challenge that must be collaboratively overcome for collaboration [93].

It is not culturally acceptable to alter the words or provide an interpretation of the words of traditional healers. The following are some notable excerpts from traditional healer interviews that occurred in the literature reviewed:

*The doctors and nurses at a local hospital asked me to speak to them on natural medicines*. *So I did*. *You could tell the doctors have a hard time trying to understand traditional healing and the use of plants to heal…it is hard for them to understand*. *Some of them got up and left when I started to talk about how you have to develop a relationship with the plant world…They sometimes have a hard time if things are not done their way…I respect the medicine*, *I just wish Western medical persons would understand* [94] …

*When we gather medicine…the plant has a spirit in it…and…the spirit of those plants stays in the medicine…Every individual is different…every remedy is different…because specific things work for specific people…We’re made up of four parts…physical*, *mental*, *emotional*, *and spiritual*. *Sometimes sickness can be caused by imbalance within a person*. *When we do Indian healing…it goes to the source of the problem…not to the symptoms* [94].

*It’s a very powerful gift that we’ve been given…I am not a healer…I am only an instrument in that whole process*. *I am the helper and the worker*, *the preparer*, *and the doer*. *The healing ultimately comes from the Creator…With the lighting of that smudge*, *holding that eagle feather while we pray…these sacred medicines*, *these sacred pipes*, *and everything that we carry in our bundles*. *That’s where the strength comes from…from those medicines*, *from Mother Earth*, *and from the Creator*… *You are a part of creation*, *you’re a part of everything…there is this interrelatedness of all things*, *of all creation*, *and everything has life…we’re a whole family*. *And we’re related to all living things and all beings and all people* [95].

*I’ve been saying it for years*. *We need more medicine people*. *We need more Native healers…male and female* [96].

It was apparent throughout the articles reviewed for this category that many traditional healers were not opposed to Western medicine; however, many had voiced concerns that Western medicine seemed to not respect them (i.e., didn’t respect their way of thinking or disregarded their knowledge base). Overall, a deep understanding and appreciation for the long-standing colonial injury felt in many Indigenous communities demonstrated through the cumulative effects of trauma ‘snowballing’ across generations [94] has become a platform for much of the traditional healers’ work in their home communities. To work with these present and historical harms, there was a clear advocacy among many of the traditional healers interviewed for ensuring the availability of therapeutic talk within cultural settings in addition to ceremonial participation to help facilitate healing and the revival of traditional spiritual beliefs [97].

## Discussion

This scoping review identified 249 articles that were predominately qualitative in nature, pertaining to traditional Indigenous medicine in the North American context. Although there was broad coverage of the topic area, it became apparent that many of the published articles were written from an ‘outsider’ perspective (i.e., observational research by scholars outside of the Indigenous communities themselves). With this, there was a slight shift noted in the type of research that was completed on traditional medicine around the 2000s. Prior to this date, it became apparent by the writing style used by many authors (i.e., they, them, etc.) that the articles were very much written “about” Indigenous people and their traditional medicine practice(s). Although post-2000 there was still quite a large volume of articles written by non-Indigenous scholars, there was an increasing presence of articles authored or co-authored by Indigenous people themselves [13,60,68,72,76]. The significance in this regard is notable as the presentation of Indigenous medicine by outside researchers often misses key cultural nuances, sometimes uses inappropriate or even insulting terminology, has a tendency to make assumptions that are not always correct (implicit bias), and presents an application or integrationist perspective that comes from what is often perceived to be a dominant Western knowledge system. As this type of ‘outsider’ scholarship serves as the foundational academic and clinical knowledge base for many of the current assumptions around traditional medicine, it was important to catalogue where some of the noted bias comes from.

Although it can be culturally inappropriate to assume there are pan-Indigenous ways of looking at traditional medicine and its practice (due to often stark differences in the practice of traditional medicine regionally), similar sentiments were expressed throughout many of the published articles. One was the assumed dominance of conventional medicine over traditional medicine practice, presented sometimes unconsciously through Western providers’ or researchers’ accounts of the subject and the language used. One possible consideration in this respect is that Indigenous-based interventions were often defined by a Western methodological approach and governance structure, which could be said to constrain and change the descriptions or programs themselves into something they were not actually meant to be. One solution to this issue would be to utilize an Indigenous methodological approach, governance structure, and reporting approach for these interventions, and then adapt the Western system to *this* approach and structure instead [58]. This would better ensure the centering of an Indigenous worldview and knowledge system through a truly self-determined Indigenous model with a potentially higher degree of success.

There is often a misperception that Indigenous peoples are in need of Westernized science in order to ‘legitimize’ our knowledge and healing systems [98]. It was clear from the literature reviewed on traditional healer perspectives that there was great opportunity for Western medicine and providers to learn about other ways of looking at health and disease in a form of respectful cooperation with Elders and Indigenous communities. This is consistent with the work of Berbman in 1973 who tells a story about a psychiatrist who brought some Navajo medicine men into his practice to demonstrate some of the things that he does in his practice [99] (i.e., the psychiatrist’s intent was to teach the medicine men). The psychiatrist demonstrated putting a Navajo woman under hypnosis for the medicine men.

One of the medicine men stated, “I’m not surprised to see something like this happen because we do things like this, but I am surprised that a white man should know anything so worthwhile… they [then] asked that my subject … diagnose something [while under hypnosis]. I objected, saying that neither she nor I knew how to do this and that it was too serious a matter to play with. They insisted that we try, however, and finally we decided that a weather prediction was not too dangerous to attempt. …When my subject was in a deep trance, I instructed her to visualize the weather for the next six months. She predicted light rain within the week, followed by a dry spell of several months and finally by a good rainy season in late summer. I make no claim other than the truthful reporting of facts: She was precisely correct” [99].

It was also evident through the articles reviewed that many Indigenous peoples using traditional medicine do not disclose this use to their Western healthcare providers. This reflects on the importance of developing culturally safe health systems and healthcare providers with strong communication skills for diverse patient settings. The story told by David Baines about the oncologist calling the patient’s traditional healer a “witch doctor” was a clear example of a lack of respect for utilizing a shared decision-making methodology for best outcomes in a clinical setting [73]. Implicit as well as overt bias against medical pluralism in diverse settings needs to be acknowledged and addressed in often authoritarian institutional settings [100,101] for best patient outcomes.

Overall, there has been a recent push with somewhat more acceptance in certain conventional medical settings towards supporting traditional Indigenous medicine interventions as demonstrated in some of the literature in this scoping review; however, the question remains whether or not “these efforts tend to represent political achievements more so than bona fide epistemological reconciliation” [72]. With continuing and significant health disparities existing in Indigenous populations in North America [102], a broader concerted effort needs to be mobilized and operationalized to ensure that Indigenous self-determined ways of knowing in relation to health and delivery of care is prioritized. Initial outcomes are promising in regard to traditional medicine’s benefit for Indigenous peoples in self-determined healthcare environments and settings. This has been clearly demonstrated by some of the literature reviewed here, yet, without more formalized support from all levels of the healthcare system, it will be difficult to expand these benefits and health outcomes to all Indigenous peoples who desire this type of care. This review and database (S2 Table) will hopefully serve as a repository for a portion of the academic literature contributing to practice, policy making, and research on this topic. This effort is aligned with Article 24 of the United Nations Declaration on the Rights of Indigenous Peoples (UNDRIP):

Indigenous peoples have the right to their traditional medicines and to maintain their health practices, including the conservation of their vital medicinal plants, animals, and minerals [1].

### Limitations

This scoping review was an attempt to catalogue the literature in the area of traditional Indigenous medicine in the North American context. The use of defined categories may give the impression of distinct traditional medicine themes unrelated to each other; however, due to the wholistic nature of traditional medicine, there will always be substantial overlap between concepts given the interconnected nature of all aspects of Indigenous healing practices. Categorical themes were used to help create some organization of the large body of literature aiding with delineating future research needs as well as for the ease of pulling for programmatic and policy needs.

It is possible, due to the substantial overlapping terminology with other fields, that some articles may have been missed in the search strategy. With this, an effective search strategy in this field would require the searcher to be familiar with how Indigenous medicine terminology is commonly used and applied in academia to be able to correctly select and screen articles from a very large databases of mixed disciplines. Traditional medicine terminology can be complex and can be referenced using other languages or simply geographic location. Due to this, any published articles that used unique ways of referencing traditional medicine or were described using an Indigenous language term could have caused additional articles to be missed; however, due to saturation being reached in the methods review, we feel the literature was well represented in our database. Regardless, this comprehensive database (S2 Table) of the available literature should not be considered exhaustive of all available material on this topic.

From an Indigenous worldview, culture and cultural practices can be looked at and examined as being a form of medicine. Even traditional language can be considered a form of cultural medicine [103]. This review excluded studies to this effect due to the variation in interpretations that are possible in this area; however, this exclusion was not intended to degrade or minimize the importance of culture as a healing strategy in any way. Due to the need to capture one defined area of this topic on traditional medicine and healing as a first step, further research can now build upon this work by evolving the scholarship area to be inclusive of all facets of Indigenous healing.

Traditionally within Indigenous communities, knowledge on traditional healing or the medicines themselves was and is passed down through a strong oral tradition that often involves deep ceremonial practice. As knowledge transmission in the North American context most often does not include a written record, historical and present-day information on community practice in this area is rightfully held within Indigenous communities themselves. This form of knowledge needs to be recognized, honored, and respected in the context of the traditional protocols that the respective community follows under the guidance of their Elders. This knowledge is the true knowledge that is most often not reflected in written academic scholarship. Some Indigenous communities have become more engaged with research as you will have seen throughout this review; however, some choose not to engage in this form of knowledge transmission for a variety of important reasons. This review, although detailed, is therefore only a small snapshot of the vast knowledge that exists within Indigenous communities in North America.

A critical review of the retained full text articles was not completed as the intent was to provide a representative and complete database on this topic. In addition, the vast array of formats and methodologies used in the Indigenous traditional medicine literature make the dominant Western metrics of validity simply not applicable to the current research purpose. Because it is not culturally acceptable to critique traditional Indigenous medicine, an Indigenous methodology was honored. Using an inclusive framework for this topic, several articles that were not written by Indigenous peoples or communities were included, which in some cases portrayed gross stereotypes from ‘outside’ observations of traditional medicine practice(s). The reader is therefore advised to exercise caution when utilizing information from ‘outside’ observational and older studies that may not be reflective of actual and current Indigenous community perspectives on the topic discussed. To this end, we highly recommend prioritizing the respectful engagement of Indigenous scholars and/or their scholarship, community members, and local knowledge holders to better ensure the concepts and resources presented here will be grounded and relevant within any local or cultural context.

## Conclusion

This scoping review identified 249 articles pertaining to traditional Indigenous medicine in the North American context with the following categorical themes being identified: General Traditional Medicine, Integration of Traditional and Western Medicine Systems, Ceremonial Practice for Healing, Usage of Traditional Medicine, and Traditional Healer Perspectives.

Although effort has been made to better accommodate Indigenous ways of knowing and healing into healthcare settings and delivery models, self-determined options for traditional Indigenous healing are still lacking in Western institutions. This scoping review underscores the crucial need to further examine the dynamics of healthcare relations in a post-colonial context, with more open spaces for dialogue surrounding the use of Indigenous traditional healing often desired in racially diverse medical settings. The prerequisite to move closer to transformative practice in this area involves prioritizing further research and communication on this topic with a focus on applied self-determined interventions and programming.

## Supporting information

S1 Checklist Preferred reporting items for systematic reviews and meta-analyses extension for scoping reviews (PRISMA-ScR) checklist. (PDF)

S1 Table Sample electronic research database search strategy (PubMed). (PDF)

S2 Table Traditional Indigenous medicine in North America article database. (XLSX)
