# Supplementary material for: Traditional Indigenous medicine in North America: A scoping review
Source: PLoS One. 2020 Aug 13;15(8):e0237531. doi: 10.1371/journal.pone.0237531 (PMC7425891; doi:10.1371/journal.pone.0237531)
Supplement: S1 Table — (PDF) [file pone.0237531.s002.pdf]

S1 Table. Sample Electronic Research Database Search Strategy (PubMed)

**Dates**	All dates to June 29, 2020
**Language(s)**	All
**Keywords**	Indian OR "American Indian" OR “Alaska Native” OR “Native American” OR “First Nations” OR Indigenous OR Métis OR Metis OR aborigin* OR "First Nation*" OR amerindian OR tribe OR tribal OR reservation OR Pima OR Cherokee OR Inuit OR Yupik OR Navajo OR Lumbee OR Apache OR Ojibw* OR Sioux OR Salish OR Aleut OR Inupiat OR Haida OR Athabaskan OR Zuni OR Cheyenne OR Hopi OR Cherokee OR Mohawk OR Crow OR Mi’kmaq OR Micmac OR Lakota OR Cree OR Blackf* OR Dene* OR Assiniboine OR Chipewyan OR Eeyou Istchee OR Eskimo OR Haudenosaunee OR Innu OR Inuk*, Inuvialuit OR Iroquois OR Lenape OR Menominee OR Nakota OR Nakoda OR Oji-Cree OR Sioux OR Dakota OR Turtle Island OR Gwitchin OR Vuntut Gwitchin OR Choctaw OR Chippewa OR Apache OR Pueblo OR Shawnee OR Cahuilla OR Coushatta OR Arapahoe OR Paiute OR Mono OR Cabazon OR Me-Wuk OR Diegueno OR Catawba OR Cayuga OR Chemehuevi OR Chickahominy OR Chickasaw OR Potawatomi OR Pomo OR Cocopah OR Coeur D’Alene OR Comanche OR Yakama OR Siletz OR Chehalis OR Coos OR Goshute OR Umatilla OR Cortina OR Coushatta OR Cowlitz OR Shoshone OR Shawnee OR Maidu OR Kumeyaay OR Yavapai OR Mojave OR Havasupai OR Ho-chunk OR Hoh OR Maliseet OR Hualapai OR Inaja OR Ione OR S’Klallam OR Jamul OR Karuk OR Keweenaw OR Kickapoo OR Kiowa OR Klamath OR Kootenai OR Luiseno OR Odawa OR Cupeno OR Elwha OR Lummi OR Makah OR Wampanoag OR Mesa OR Miccosukee OR Moapa OR Modoc OR Monacan OR Maidu OR Morongo OR Muscogee OR Narragansett OR Nez Perce OR Nisqually OR Nooksack OR Oneida OR Onondaga OR Osage OR Paiute OR Luiseno OR Pamunkey OR Pascua* OR Passamaquoddy OR Pawnee OR Peoria OR Picayune OR Creek* OR Ponca OR Puyallup OR Quapaw, Quechan OR Quinault OR Rappahannock OR Sac & Fox Nation OR Sac and Fox Nation OR Samish OR Chumash OR Seminole* OR Seneca OR Shinnecock OR Shoalwater OR Oyate OR Snoqualmie OR Ute OR Squaxin OR Suquamish OR Kumeyaay OR Tejon OR Muscogee OR Osage OR Thlopthlocco OR Tohono O’odham OR Tonkawa OR Tulalip OR Tule River OR Tuscarora OR Keetoowah OR Skagit OR Wampanoag OR Washoe OR Wichita OR Winnebago OR Winnemucca OR Wyandotte OR Yavapai* OR Yocha* OR Yurok OR Zuni OR Yellowknives OR Sarcee OR Wasagamack OR Anishinabe OR Maliseet OR Tli* OR Mississauga OR Odawa OR Oneida OR Tuscarora OR Oji* OR Assiniboine OR Tlingit OR Tagish OR Tutchone OR Elders<br>**AND**<br><br>“Traditional healing” OR “Traditional healer” OR healing OR religion OR “Medicine m*” OR “Medicine wom*” OR “Sweat lodge” OR “Traditional Medicine” OR Ceremonial tobacco OR Ceremon* OR Shaman* OR “Traditional medicine policy”
